# Wireless Sensor Networks for Noise Measurement and Acoustic Event Recognitions in Urban Environments

**DOI:** 10.3390/s20072093

**Published:** 2020-04-08

**Authors:** Liyan Luo, Hongming Qin, Xiyu Song, Mei Wang, Hongbing Qiu, Zou Zhou

**Affiliations:** 1 Provincial Ministry of Education Key Laboratory of Cognitive Radio and Signal Processing, Guilin University of Electronic Technology, Guilin 541004, China; xiaoyan12027@163.com (L.L.); qhm1702303041@163.com (H.Q.); songxiyu@guet.edu.cn (X.S.); qiuhb@guet.edu.cn (H.Q.); zhouzou@guet.edu.cn (Z.Z.); 2 Guangxi Key Laboratory of Wireless Broadband Communication and Signal Processing, Guilin University of Electronic Technology, Guilin 541004, China; 3 College of Information Science and Engineering, Guilin University of Technology, Guilin 541004, China

**Keywords:** WASNs, noise measurement, acoustic events recognition, CNNs, real-life scenarios

## Abstract

Nowadays, urban noise emerges as a distinct threat to people’s physiological and psychological health. Previous works mainly focus on the measurement and mapping of the noise by using Wireless Acoustic Sensor Networks (WASNs) and further propose some methods that can effectively reduce the noise pollution in urban environments. In addition, the research on the combination of environmental noise measurement and acoustic events recognition are rapidly progressing. In a real-life application, there still exists the challenges on the hardware design with enough computational capacity, the reduction of data amount with a reasonable method, the acoustic recognition with CNNs, and the deployment for the long-term outdoor monitoring. In this paper, we develop a novel system that utilizes the WASNs to monitor the urban noise and recognize acoustic events with a high performance. Specifically, the proposed system mainly includes the following three stages: (1) We used multiple sensor nodes that are equipped with various hardware devices and performed with assorted signal processing methods to capture noise levels and audio data; (2) the Convolutional Neural Networks (CNNs) take such captured data as inputs and classify them into different labels such as car horn, shout, crash, explosion; (3) we design a monitoring platform to visualize noise maps, acoustic event information, and noise statistics. Most importantly, we consider how to design effective sensor nodes in terms of cost, data transmission, and outdoor deployment. Experimental results demonstrate that the proposed system can measure the urban noise and recognize acoustic events with a high performance in real-life scenarios.

## 1. Introduction

In urban environments, human activities such as transportation [1], industrial manufacturing [2], and building construction [3] lead to an increase in noise pollution, which poses a distinct threat to people’s health [4] and quality of life [5]. In order to alleviate these negative effects, some methods are proposed in recent years to measure urban noise using sound level meters. 

Noise is traditionally measured using sound level meters that require a professional operation and expensive cost. Instead, the combination of numerous smartphones acting as sound level meters allows achieving large-scale monitoring. Different algorithms such as Fourier-based algorithm, time domain-based algorithm, and normalized time domain algorithm were performed on multiple smartphones to measure noise levels [6,7,8], which present the possibility to measure noise levels with an accuracy degree comparable to professional meters. Nevertheless, the trouble of manual operation becomes a difficulty when it comes to the demand for longtime measurements. With the development of IoT, the Wireless Acoustic Sensor Networks (WASNs) have become a promising resolution to address these challenges. In [9], a WASN is being deployed in Ostrobothnia (Western Finland) in order to measure acoustic noise. In addition, the project [10] designed for the monitoring of the traffic noise in Xiamen City was used to capture the traffic noise data and the researchers then model these data to simulate the traffic of roads. The well-known project, DYNAMAP [11], aiming to develop a dynamic noise mapping system can detect and represent the acoustic impact of road infrastructures using customized sensors and communication devices in real time. In the framework of the LIFE DYNAMAP project [12], two WASNs have been deployed in two pilot areas to measure the noise levels detected by the sensors, which are generally used to scale basic noise maps stored in a database and processed on a general GIS platform. Moreover, other research groups have been also devoted to this project to promote the progress [13,14]. More details can be found in [15], which reviews the most relevant WASN-based approach developed to date focused on environmental noise monitoring. Although much forward progress on noise measurement and mapping have been achieved, these projects still face the difficulty when it comes to the analysis of noise source. To address these problems, some projects consider using the combination of environmental noise measurement and acoustic events recognition for the analysis of noise pollution. The distributed sensor network allows for measuring different noise level parameters and classifying among various acoustic scenes and events using the Convolutional Neural Networks (CNN) in [16]. The data captured by the sensor network was transferred to a central server for data post-processing and a web-based application allows for various real-time visualization of noise distribution. The SONYC project [17] deployed 56 low-cost acoustic sensors across New York City to monitor urban noise and perform a multilabel classification of urban sound sources in real time. Obviously, the combination of environmental noise monitoring and acoustic events recognition are of great significance to analyze the noise pollution and the quality of living environments, raising civic consciousness and formulating corresponding noise reduction strategies.

Although previous works on noise assessments and acoustic event recognitions achieve significant progress, they remain impractical in complex urban environments due to the following drawbacks:

(1) The large-scale acoustic sensor networks, especially those deployed for acoustic events recognition, generate a large amount of data from each sensor node, which brings the large pressure on transmission of network.
(2) The sounds of interest are superimposed to a significant level of background noise in real-life scenario, which might have an infaust influence on the accuracy of recognition.
(3) The limitation of long-term outdoor monitoring: (1) The dependence of the sensor nodes on the restricted power supplies (batteries) is a defect, as it impedes the longevity of the network; (2) the waterproof measures of sensor nodes are not enough to be resistant to extreme outdoor weather.

Most importantly, the development of acoustic signal processing techniques to identify noise events based on reliable WASNs in urban areas is an open and new challenge [15]. To address this challenge, this paper presents a complete conceptual implementation based on the Wireless Acoustic Sensor Network (WASN) for recording, transferring, post-processing, and recognition of noise in order to create and visualize noise maps of acoustic events, as well as to present additional information and noise statistics. To address these drawbacks mentioned above, the proposed system mainly includes the following characteristics:

(1) Every acoustic sensor node in our system is powered by a solar panel and is waterproof with the package, which is well-suitable for long-term outdoor monitoring. Of course, it can also be powered by a directly available power if the outdoor power is possible.
(2) The combination of endpoint detection and compression with the Adaptive Differential Pulse Code Modulation (ADPCM), which is performed on the embedded processing board, makes it efficient to reduce the amount of data.
(3) The acoustic database is made up of live recordings created by sensor nodes, which is helpful in improving recognition accuracy.
(4) A novel monitoring platform is designed to intuitively present noise maps, acoustic event information, and noise statistics, which allows users to conveniently get information on the noise situation.

Specifically, the proposed system utilizes sensor nodes equipped with various hardware devices such as acoustic sensors, processing boards, and transmission modules to capture the urban noise level, record the acoustic events, and transfer the resulting data to the remote server. Such a server would be used to complete data reception, storage, feature extraction, recognition, as well as monitoring platform construction. We conduct some experiments of sensor nodes and deploy a WASN to process the audio data collected by 50 sensor nodes on campus to validate the performance of the system.

The rest of the paper is organized as follows. Section 2 introduces the hardware and software used in the proposed system. Section 3 describes the implementation steps of our system. Section 4 presents the deployment of the proposed system, experimental results, and analysis. Finally, Section 5 concludes the paper.

## 2. Design Details of the Proposed System

**Figure 1:**
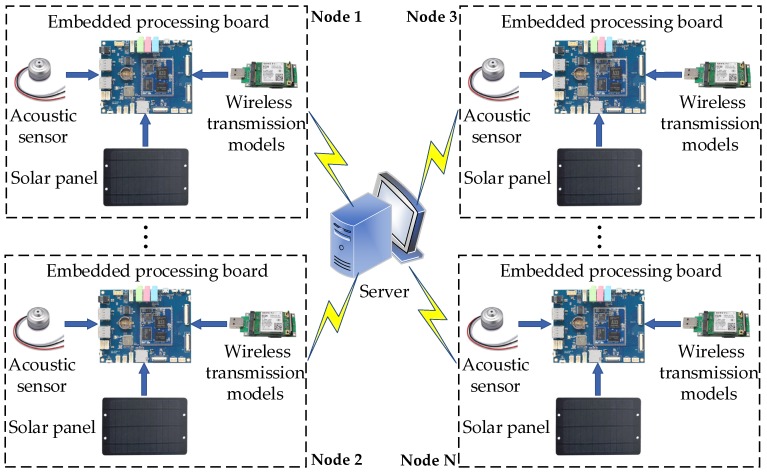
The overview of the proposed system.

The overview of the proposed system is shown in Figure 1. Here, acoustic sensors are used to collect sound signals in urban environments. Then, these signals are processed by embedded processing boards through endpoint detection and the ADPCM method and are transferred to the server using wireless transmission modules. Finally, the remote server aggregates and stores the resulting data from multiple nodes for further analysis such as the feature extraction and event recognition.

### 2.1. Hardware Details

(1) Acoustic sensor: Since the frequency range of sound audible to the human ear is 20–20 kHz and there is a large background noise in the outdoor environment, we prefer to select the sensor from the two aspects of frequency response and noise reduction. The sensor has the frequency range of 20–20 kHz and the maximum monitoring area of 80 square meters. The sensor has an amplification circuit, filter circuit, and noise reduction circuit inside, which can effectively filter out background noise.
(2) Embedded processing board: Based on the low cost and the slow power consumption, we chose the extra processing board with a rich audio interface and high-performance audio encoder to improve audio quality. The board is paired with the A33 processor which uses a quad-core CPU (CortexTM-A7: Advanced RISC Machines, Cambridge, UK) and a GPU (Mali-400 MP2: ARM Norway, Trondheim, Norway) at frequencies up to 1.2 GHz and has 1 GB DRAM and 8 GB FLASH. In addition, it allows users to expand the storage space by using SD cards.
(3) Wireless transmission module: Adopting the 3GPP Rel-11 LTE technology, the Quectel-EC20 4 G module delivers 150 Mbps downlink and 50 Mbps uplink data rates. A rich set of Internet protocols, industry-standard interfaces, and abundant functionalities extend the applicability of the module to a wide range. The module also combines high-speed wireless connectivity with an embedded multi-constellation high-sensitivity GPS + GLONASS receiver for positioning.
(4) Solar panel: Since our system is used for the long-term outdoor monitoring, we chose the solar panel with a battery and a power controller inside. During the day, the solar panel absorbs energy to charge the battery, and supplies power to the embedded processing board at the same time. It is only used to power the processing board at night. In addition, the solar panel we chose is embedded with a 5 V and 10,500 mAh battery, which allows the processing board to work continuously about two days without sunshine.

Table 1 shows the hardware costs.

### 2.2. Software Details

The embedded processing board we chose is based on Android, so we chose the Android Studio as the development platform for APP. The Android Studio is an Android Integrated Development Environment (IDE) from Google that provides integrated Android development tools for development and debugging. The software we installed on the server is Eclipse, MySQL, PyCharm, TensorFlow, and Sublime Text. Eclipse is an open, extensible IDE not targeted for any specific language or environment, which is used to develop an application to receive audio data from the embedded processing board. MySQL is a relational database management system for the storage and management of audio data. PyCharm and TensorFlow are used to build convolutional neural networks to recognize acoustic events. Sublime Text is an advanced code editor for the construction of the monitoring platform.

## 3. System Implementation

**Figure 2:**
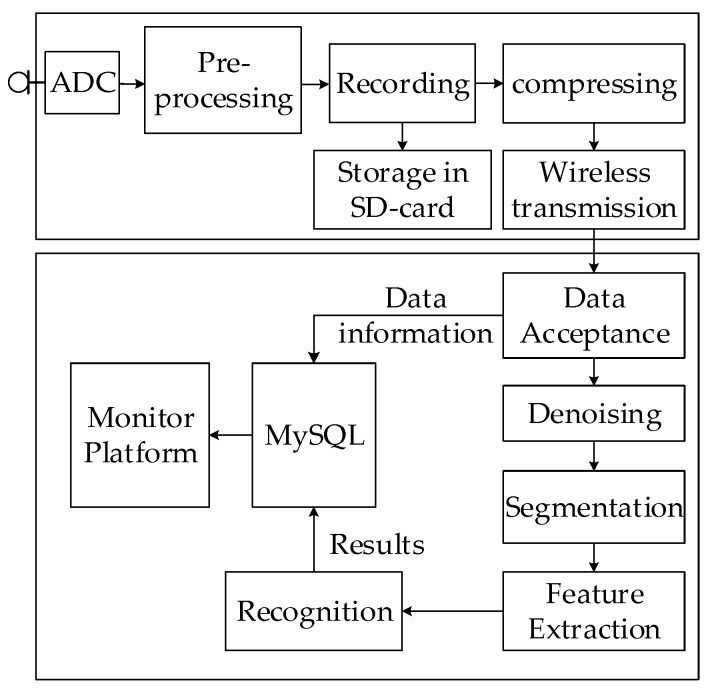
The block diagram of the proposed system.

The block diagram of the proposed system is shown in Figure 2. The proposed system has six implementation stages, which are: (1) sampling and recording; (2) compress and transmit; (3) receive and storage; (4) feature extraction; (5) classification; (6) monitoring platform. We describe each of these in detail in the following sections.

### 3.1. Sampling and Recording

The aim of this step is to measure the sound levels and obtain the audio files. The frequency range of sound audible to the human ear is 20–20 kHz [18], so we use a sample with 44.1 kHz, quantization with 8-bit, and coding with PCM to capture sounds. A-weighting is now commonly used for the measurement of environmental noise and industrial noise among the five different frequency weightings (called A, B, C, D, and Z) for measuring the sound pressure level [19]. The A-weighted equivalent sound level LA_eq,T_ [20,21] is given by:

(1) $${\mathrm{LA}}_{\mathrm{eq},T}=10\log_{10}\left(\frac{1}{T}\int_{0}^{T}{\left(v_{A}(t)\right)}^{2}dt\right)+\Delta$$

where v_A_(t) is the result of passing the induced voltage in the microphone through an A-weighting filter [22] and Δ is a constant offset determined by calibrating the microphone against a standard sound level meter. The measurement of noise levels is a continuous process of real-time implementation, but the recording is not continuous. Only when the level reaches a threshold will it be triggered. The delay of the device itself may result in the loss of audio samples, thus we chose the data point in the buffer for 500 ms before the trigger moment as the beginning of recording to solve the problem. To reduce the amount of data, an endpoint detection algorithm based on the sub-band selection spectral variance [23,24] is adopted in this work. We can discriminate the useful segment to be recognized from the noisy signal according to the variation of the spectral variance. The sub-band spectral variance is expressed as follows:

(2) $$X_{i}=\left\{X_{i}(1),X_{i}(2),\cdots ,X_{i}\left(\frac{N}{2}+1\right)\right\}$$

where *X_i_* is the amplitude of the *i*-th frame, and the length of each frame is N.

(3) $$M_{i}(m)=\sum_{k=1+(m-1)p}^{1+(m-1)p+(p-1)}\left|X_{i}(k)\right|$$

where *M_i_(m)* is the magnitude of the m-th band of the *i*-th frame, and m is the sub-band index, p is the spectral line number in each sub-band.

(4) $$Ei=\frac{1}{q}\sum_{k=1}^{q}XX_{i}(m)$$

where *E_i_* is the mean value of the sub-band of the *i*-th frame, and q is the number of the sub-band.

(5) $$D_{i}=\frac{1}{q-1}\sum_{k=1}^{q}{\left[XX_{i}(m)-E_{i}\right]}^{2}$$

where *D_i_* is the sub-band spectral variance of the *i*-th frame.

The experimental results are introduced in Section 4. After sampling and recording, the data mentioned above is saved in the SD card and transferred to the remote server for further processing.

### 3.2. Compress and Transmit

To further reduce data capacity, the recording data is compressed before transmission. An effective way to compress audio data is using the Differential Pulse Code Modulation (DPCM) that encodes the analog audio input signal using the difference between the current and the previous sample [25]. The difference can be large or small because the sound signal is random. To accommodate it, ADPCM is more applicable [26]. In this work, we implemented the ADPCM on the embedded processing board. The influence of compression with ADPCM on audio quality and recognition will be reported in later chapters. After compression, a packet including audio, noise level, triggered time of recording, coordinates, and device number is transferred to the server through the 4 G wireless module.

### 3.3. Receive and Storage

Whenever the server receives a packet, it decompresses the packet to the appointed path of storage to get the original data. As shown in Table 2, the triggered time (Time), the location of the node (Coords), the identification code of the node device (IMEI), and the noise level (Decibel) are stored in MySQL for efficient management. Especially, the GPS devices submit data in the form of sentences, which are defined by the NMEA-0183 protocol. There are lots of sentences in the NMEA protocol and it is infeasible to save the whole emitted information in a database, therefore we are going to deal with only the $GPRMC sentence, which contains the necessary minimum information for location purposes, as can be seen in Appendix A. After obtaining the coordinates of latitude and longitude which can be extracted from the $GPRMC sentence, we can convert the coordinates to the corresponding map coordinates by calling the API interface of Baidu Map. Finally, we save the map coordinates in MySQL for a direct call in the monitoring platform. The monitoring platform automatically accesses these data to update the information displayed on the platform.

### 3.4. Feature Extraction

**Figure 3:**
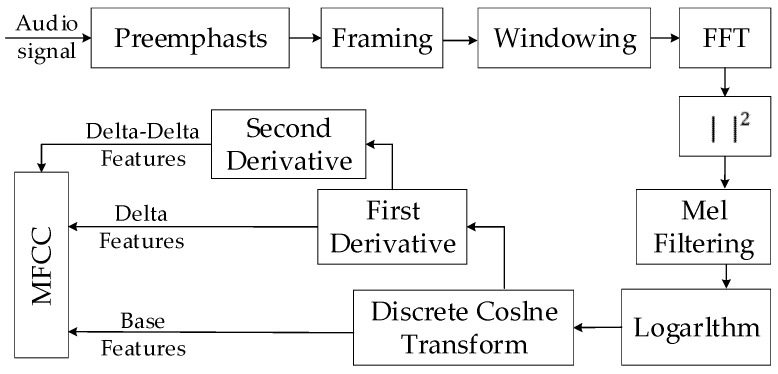
The process of feature extraction.

The MFCC-based acoustic features have been proven effective in detecting various special audio events [27]. MFCC is used as the feature extraction method in this work. The process of extracting the MFCC-based acoustic features is shown in Figure 3. It can be summarized as follows:

(1) Frame the signal into short frames. An audio signal is constantly changing, so to simplify things we assume that on short time scales the audio signal does not change much. We frame the signal into 25 ms frames. The continuous audio signal is blocked into frames of *N* samples, with adjacent frames being separated by M. We take the value of M as a half of N.
(2) Calculate the power spectrum of each frame by the following:

(6) $$S_{i}(k)=\sum_{n=1}^{N}s_{i}(n)h(n)e^{-j2\pi kn/N}\;1\leq k\leq K$$

where h(n) is an *N* samples long analysis window, and *K* is the length of the DFT. $s_{i}$(n) is the time domain signal where *n* ranges over *1-N* and *i* ranges over the number of frames. The power spectrum of the *i*-th frame is given by:

(7) $$P_{i}(k)=\frac{1}{N}{\left|S_{i}(k)\right|}^{2}$$

(3) Apply the mel filterbank to the power spectra and sum of the energy in each filter. In this step, we calculate the mel frequency by:

(8) $$f_{mel}=2595\log_{10}\left(1+\frac{f}{700}\right)$$

where *f_mel_* is the mel frequency and *f* is the frequency value. The suggested number of filterbank size K is in the range of 20 to 40, and we chose 26 because it is the standard.

(4) Take the logarithm of all filterbank energies.
(5) Take the DCT of the logarithmic filterbank energies. We keep the DCT coefficients from 2 to 13 and discard the rest.
(6) Obtain delta and delta-delta features by the derivative. The following formula is used:

(9) $$d_{t}=\frac{\sum_{l=1}^{L}l\left(c_{t+l}-c_{t-l}\right)}{\sqrt{2\sum_{l=1}^{L}l^{2}}}$$

where $d_{t}$ is the delta coefficient of *t*, and $c_{t}$ represents the cepstrum coefficient of t, and a typical value for L is 2. Delta-delta coefficients are calculated in the same way, but they are calculated from the deltas, not the static coefficients.

(7) The MFCC consists of base features, delta features, and delta-delta features.

### 3.5. Recognition

**Figure 4:**
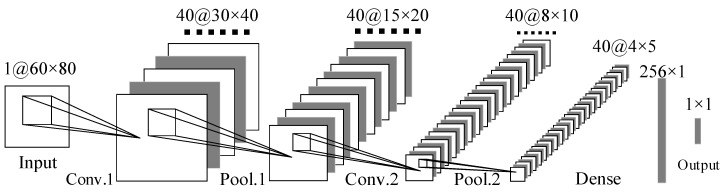
The architecture of deep convolutional neural networks (CNNs).

The ability of deep Convolutional Neural Networks (CNNs) to learn discriminative spectro-temporal patterns makes them well suited to environmental sound recognition [28]. This study refers to the classic convolutional neural network structure to design a deep convolutional neural network, as shown in Figure 4. It is comprised of two convolutional layers interleaved with two pooling layers for feature extraction, followed by one fully connected (dense) layer for deep feature integration and complex function modeling. 

Given the input $X_{\mathrm{mfcc}}$, the network is trained to learn the parameters Θ of a composite nonlinear function ℱ(⋅|Θ), which maps X to the output (prediction) Z:

(10) $$Z=ℱ\left(X_{\mathrm{mfcc}}|\Theta \right)=f_{L}\left(\cdots f_{2}\left(f_{1}\left(X|\theta_{1}\right)|\theta_{2}\right)|\theta_{L}\right)$$

where each operation $f_{\ell }\left(\cdot |\theta_{\ell }\right)$ is referred to as a layer of the network, with L = 6 layers in our proposed architecture. The convolutional $Z_{\ell }$, ℓ∈ {1, 2}, are expressed as:

(11) $$Z_{\ell }=f_{\ell }\left(X_{\ell }|\theta_{\ell }\right)=h\left(W*X_{\ell }+b\right),\;\theta_{l}=[W,b]$$

where *h(·)* is a point-wise activation function, W is a collection of 3D kernels, $X_{\ell }$ is a three-dimensional input tensor, ∗ represents a valid convolution, and b is a vector bias term. The max-pooling layers are applied after each convolutional layer ℓ∈ {2,4}, which reduces the dimensions of the output feature maps and consequently speeds up training. The final layer, ℓ∈ {5}, is fully connected (dense) and consists of a matrix product rather than a convolution:

(12) $$Z_{\ell }=f_{\ell }\left(X_{\ell }|\theta_{\ell }\right)=h\left(WX_{\ell }+b\right),\;\theta_{\ell }=[W,b]$$

where $X_{\ell }$ is flattened to a column vector of length *N*, *W* has a shape (256,800), b is a vector of length *M*, and *h(·)* is a point-wise activation function. 

Finally, recognition is completed in the fully connected layer.

### 3.6. Monitoring Platform

We design a monitoring platform for environmental noise and sound events, including noise map, environmental sound event information, and noise statistics. For the noise map, we use different color circles to represent the noise levels of sensor nodes, so that we can intuitively understand the noise situation of the monitoring areas. The noise map is updated automatically with the environmental condition changes. For the sound event information, when the sound event occurs, which can be detected by sensor nodes, the corresponding landmarks will throb briefly on the map, and the information including location, event, and time will be recorded and displayed. For the noise statistics, we show the noise variation of the monitoring area in a day. We can choose different monitoring areas to view the corresponding variation of noise. All the above data can be reviewed by selecting the date and time. 

## 4. Results and Analysis

We conduct experiments in two steps to evaluate the performance of the proposed system. First, we evaluate the performance of the sensor node, mainly related to sound acquisition and recording. Second, we evaluate the performance of the proposed system based on WASN that consists of 50 sensor nodes, mainly related to the recognition and monitoring platform.

### 4.1. Node-Based Analysis

#### 4.1.1. Packaging for the Outdoor Deployment

**Figure 5:**
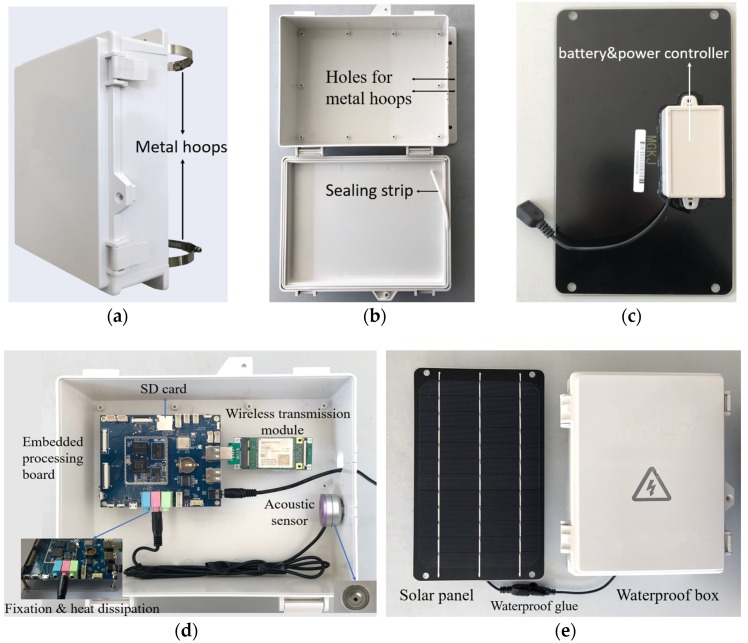
The structure of a sensor node. (**a**) External appearance of the waterproof box; (**b**) interior view of the waterproof box; (**c**) the solar panel with a battery and power controller inside; (**d**) the connection of different parts inside the box; (**e**) a whole sensor node.

In order to resist the extreme outdoor weather, the packaging of each sensor node is necessary. A waterproof box with an Acrylonitrile-Butadiene-Styrene (ABS) material, which can reach the waterproof level of IPX65 was selected. Several hoops in the back of the box are used for erect pole installation with two metal hoops, as shown in Figure 5a. A sealing strip inside the box achieves further waterproofing, as shown in Figure 5b. The battery and power controller are packaged in the back of the solar panel, as shown in Figure 5c. The different parts were all connected and placed inside the box and then connected with the solar panel, as shown in Figure 5d. The packaging of a sensor node is shown in Figure 5e. Such packaging makes it more effective in water tightness and solarization with convenient installation in the outdoor environment. 

#### 4.1.2. The Accuracy of the Sound Level

**Figure 6:**
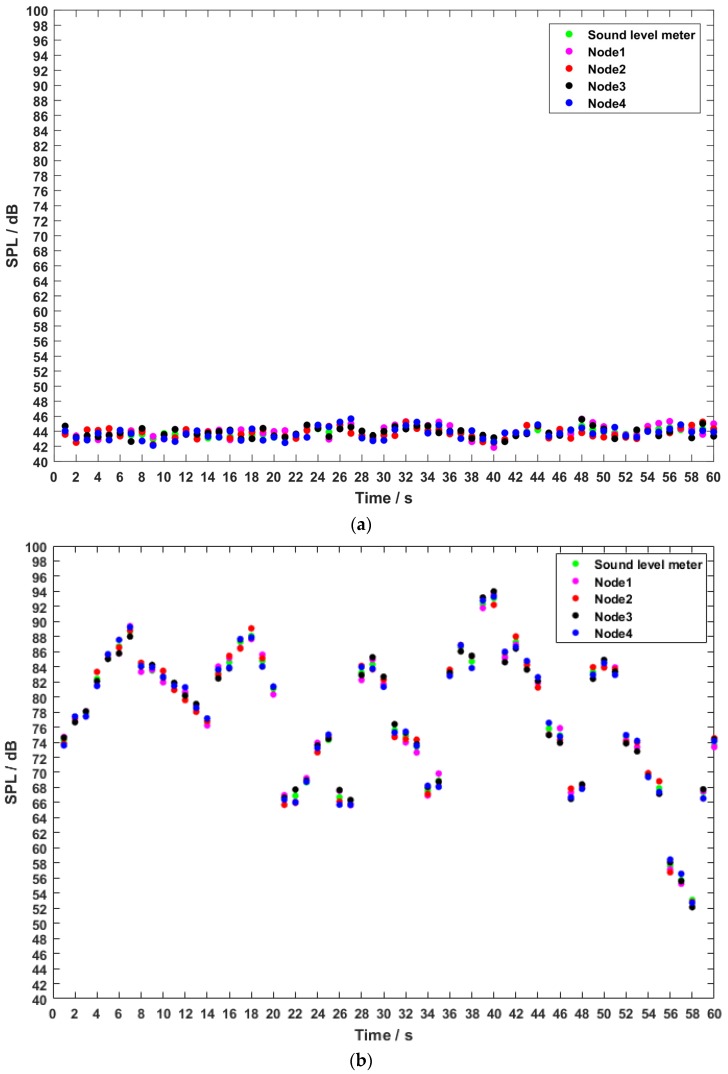
Data obtained from the sound level meter and the sensor nodes in the field test. (**a**) In the school road (low Signal to Noise Ratio (SNR)); (**b**) in the school gate (high SNR).

The test for checking accuracy and robustness is necessary since the measuring algorithm of sound level was implemented in each sensor device. Generally, the devices have minor differences on performance even if they are the same type. In addition, different environments have different effects on the evaluation of the algorithm. Therefore, we should use a professional sound level meter and multiple devices for contrastive test in complex environments with different SNRs. To sum up, a test in real-life scenarios was carried out with the professional sound level meter AWA5688 that can record the current sound level in every second [29]. We programed our devices to record the sound level every second as the sound level meter. We deployed the sound level meter and four nodes at the school road and school gate with the same position and same height to implement a 1-min test. As can be seen in Figure 6a, the variation of noise within 1 min is slight in the quiet environment of the school street. On the contrary, the variation is random and varied in the noisy environment of the school gate. In summary, the maximum error between the sound level meter and the four nodes is 0.47 dB in a quiet environment and is 0.93 dB in a noisy environment. We considered that the noisy environment with a low SNR leads to the error increase but was less than 1 dB. As such, this test indicates that the proposed system has a high accuracy of the sound level.

#### 4.1.3. The Quality of Recording Data

**Figure 7:**
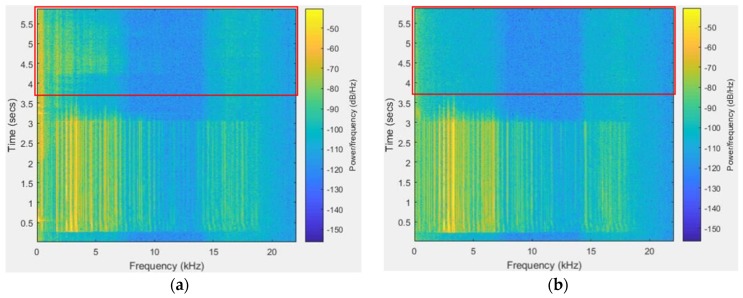
The spectrogram of the sound. (**a**) The spectrogram of the sound we recorded by the sensor in a box; (**b**) the spectrum of the sound we recorded by the sensor towards the outside of the box.

Reduction of reverberation. The acoustical attenuation of the package and the reverberation inside the package are the dominating interference of recording. To avoid the negative influence on the sensor signal, we firm the acoustic sensor towards the outside of the box through the hole, which allows the acoustic sensor to capture directly the arriving signal, as can be seen in Figure 5d. For the same sound source, we used a device with the sensor in a box and another device with the sensor towards the outside of the box to record it. As shown in Figure 7a, there is reverberation during the time of 3.8–5.8 s, on the contrary, there is no reverberation in Figure 7b. Obviously, the reverberation is effectively reduced by placing the sensor towards the outside of the box.

**Figure 8:**
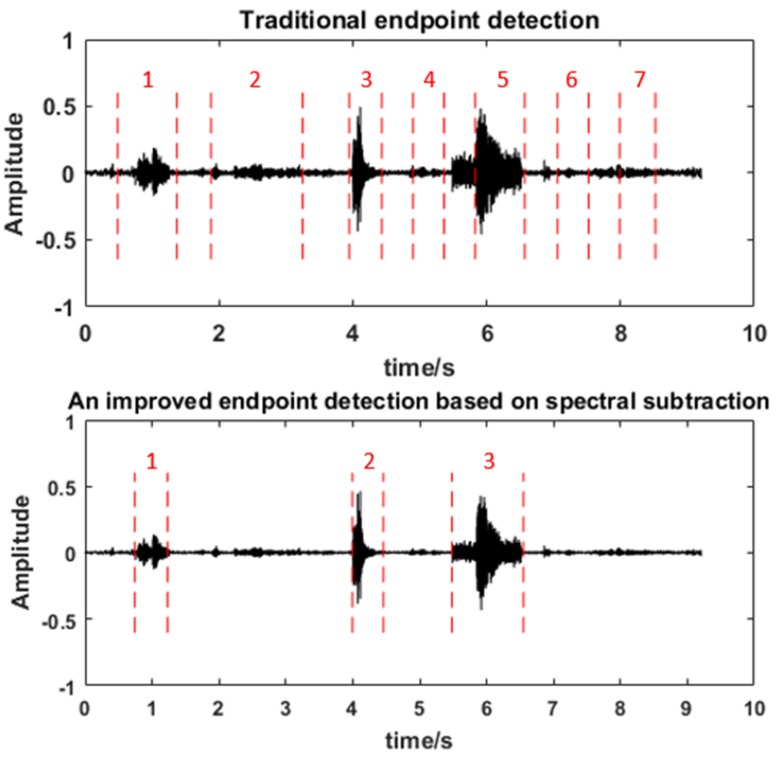
The validity of endpoint detection used in the system.

Endpoint detection. The background noise in the real environment are inevitably recorded by sensor nodes. To reduce the amount of data, we adopt the method of endpoint detection to choose useful sounds to be recognized. Referencing [23,24], we perform an endpoint detection algorithm based on the sub-band selection spectral variance on the embedded processing board. As shown in Figure 8, an audio data recorded by the system is divided into seven segments and four of them (2,4,6,7) are background noise using traditional endpoint detection. Significantly, it is divided into three segments and all of them are useful sounds using the improved endpoint detection, which presents better robustness under different SNR conditions.

**Figure 9:**
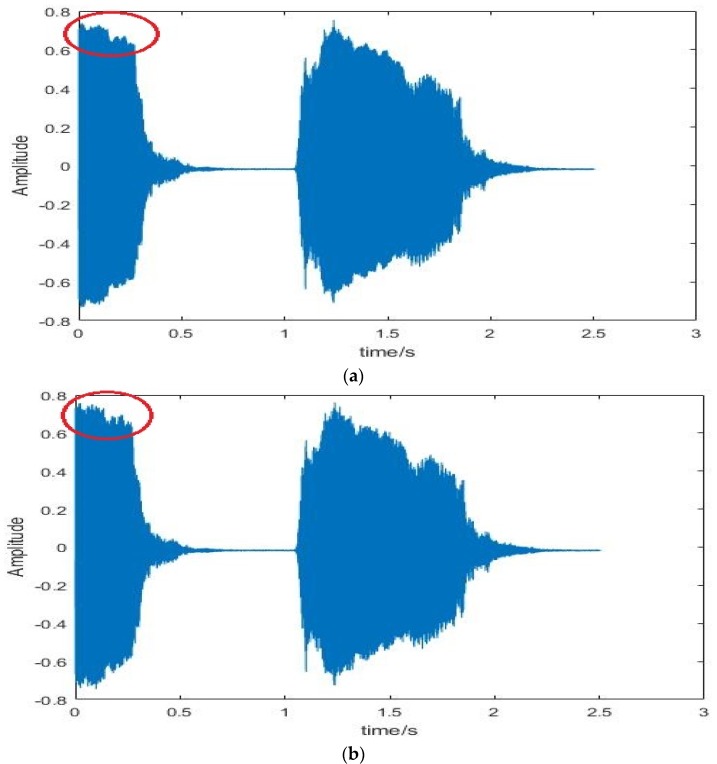

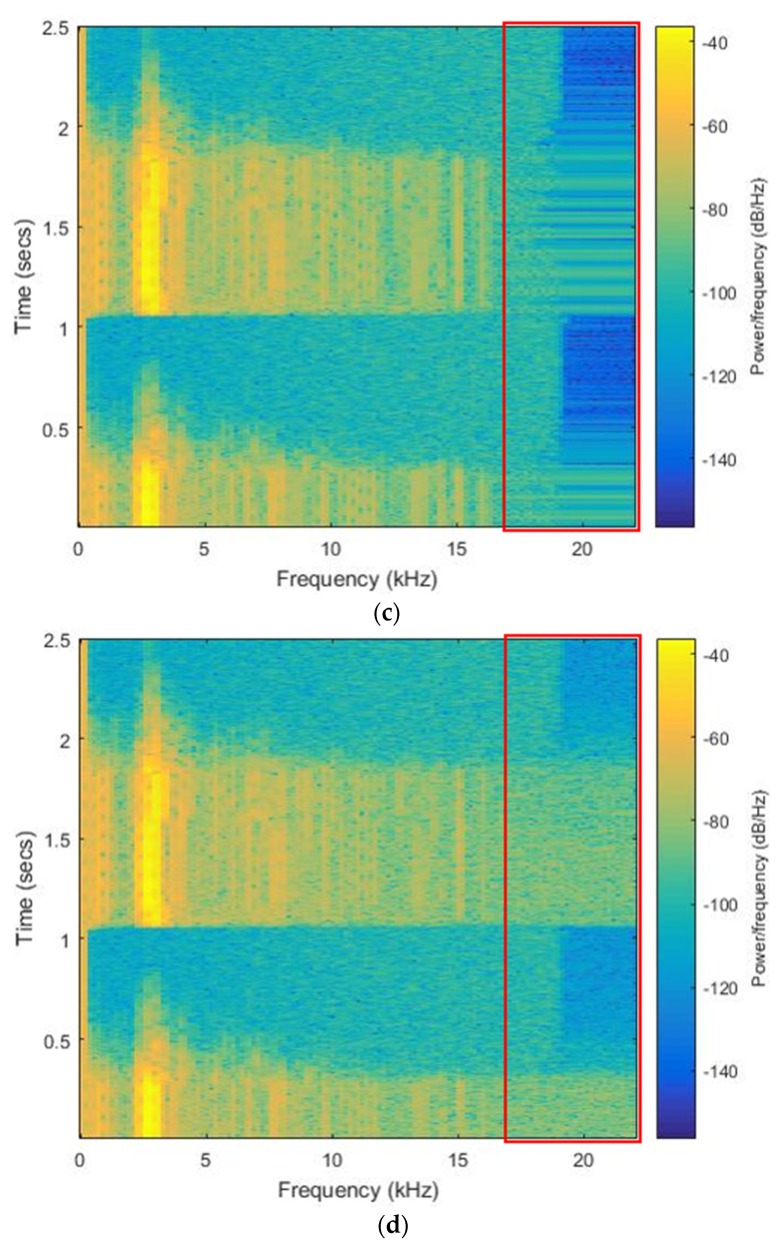
The influence of compression with the adaptive differential pulse code modulation (ADPCM). (**a**) The waveform of car horn before compression; (**b**) the waveform of car horn after compression; (**c**) the spectrograms of car horn before compression; (**d**) the spectrograms of car horn after compression.

*Compression with ADPCM.* Compression reduces the capacity but also the quality of audio, so we compare the waveforms and spectrograms before and after compression to explore the influence of compression on the sound signal. The Adaptive Differential Pulse Code Modulation (ADPCM) is a compression algorithm for 16 bits of sound data. It transforms 16-bit to 4-bit per sampling for the compression ratio of 1:4, but reduces the precision of the signal. In other words, the waveform of the signal will become blurred or blurr (spiculate) but without distortion after compression, as can be seen in Figure 9a,b. However, the compression mainly affects the frequency range of 16–22 K, which has a small impact on feature extraction and classification, as can be seen in Figure 9c,d. We will show the recognition accuracy in the next chapter.

#### 4.1.4. The Reliability of Transmission

We implement a daemon for activities in Linux, which is intended for recovery activities (measurement, recording, transmission, etc. in this work) in parallel with the transaction execution when something goes wrong in the system. In addition, in order to prevent the overload of memory, we employ the memory recovery mechanism to a termly clean buffer stock. To test the reliability of transmission, we chose 3000 car horns which are different in length (1–3 s) for the test. A phone was used to randomly play a car horn every 1 min and four sensor nodes were used to record and transmit audio data. The experiment lasted about two days and we checked the number of data received on the server after the test. The results are as follows:

The Transmission Control Protocol (TCP) that contains the retransmission mechanism was adopted in the proposed solution, resulting in no packet loss. However, the TCP may be invalid if the network is fluctuant or none, which means that the data cannot be transferred to the server. As can been seen in Table 3, several data were missed in the transmission of each node, but the missing rate is less than 0.1%, which is acceptable and reasonable for the proposed system.

### 4.2. Performance Analysis of the CNN in this Work

Traditional classifiers such as RF, KNN, and SVM can achieve good results when solving simple or completely constrained functions, but have limited capabilities of modeling for complex signals. However, the deep CNN has powerful capabilities of feature extraction and capabilities of modeling for complex signals [30]. In order to verify the classification performance of the CNN designed in this work, we compared its accuracy with traditional classifiers using the acoustic dataset published on the Internet. The Google Audio Set [31], an open environmental sound dataset, is the largest sound dataset with the most abundant sound categories. We selected three kinds of acoustic events (shots, screams, and car horns) from this dataset, and the number of sound samples for each category was 950. We divided them as 70% training, and 30% test set for each category to make an experiment of classification, and the results are as shown in Table 4. 

As can be seen from Table 4, for each category, the method of CNN presents a higher accuracy than the other classification methods. On average, the accuracy of CNN is 13.7%, 14.4%, and 18.4% higher than that of RF, KNN, and SVM, which presents that the excellent ability of CNN designed in this work makes it well suited to the recognition of acoustic events. In real-life scenarios, it is inevitable that the sounds of interest are superimposed to a significant level of background sounds, thus it might be negative to the recognition. It is necessary for us to verify the performance of the CNN in real-life applications, as explained in the following chapters.

TensorFlow, an open and stable source, is used to construct the CNN in the proposed system. In order to verify the reliability and efficiency of CNN, we performed the feature extraction and recognition with 10,000 samples of acoustic sounds and calculated the consuming time. The results are as shown in Table 5.

As can be seen in Table 5, the average time for feature extraction is 182 ms and the average time for recognition is 0.23 ms. Most importantly, the feature extraction is an offline procedure and the recognition is online, thus we consider that 0.23 ms is enough for online conduction with high efficiency.

### 4.3. WASN-Based Analysis in Real-Life Scenarios

**Figure 10:**
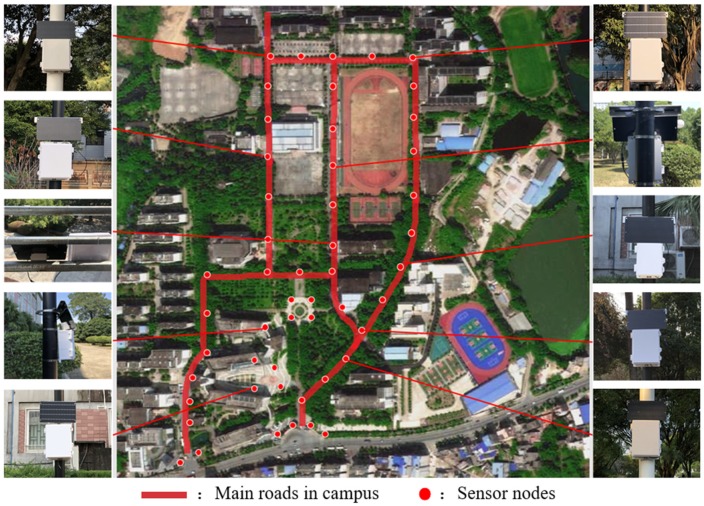
Example of the place where the sensor nodes are being deployed.

We deployed a WASN consisting of 50 sensor nodes to monitor environmental noise and acoustic event in the campus. As shown in Figure 10, these sensor nodes were deployed at main roads, teaching buildings, and gates, and we show the actual deployment of ten nodes. It should be specially explained that all the nodes were fixed on lamp poles or rails by metal hoops. In addition to the noise monitoring, it is significant for us to choose car horn as the acoustic event because the campus is a forbidden area. All data were transmitted to the server and saved in the database.

#### 4.3.1. The Recognition Accuracy Before and After Compression

To explore the influence of ADPCM on recognition, we used raw data with PCM and compressed data with ADPCM for the test. We manually selected 1742 car horns and 1579 background noises from the database to build our dataset that occupies the capacity of 511 M. We divided the dataset as 70% training, and 30% test set for each species, as shown in Table 6. The recognition result is shown in Table 7. Next, we compressed the data of the dataset by the ADPCM method, and the compressed dataset occupies the capacity of 116 M. We used the compressed dataset to test again with the same conditions, and the recognition result is shown in Table 8.

The recognition precision of the car horn is 93.8% (491/(491+32)×100%) in Table 7 and is 91.8% (480/(480+42)×100%) in Table 8. It is reduced by 1.9% after the ADPCM compression, which was considered as a small influence on recognition. In addition, the capacity of raw dataset was about four times as big as the capacity of compressed dataset. Obviously, the compression with ADPCM reduces the capacity of transmission while ensuring the high recognition accuracy, thus we considered it as suitable for our system.

#### 4.3.2. The Recognition Accuracy of Audio Streaming

A reliable car horn detection algorithm has to make a decision whether a car horn has occurred based on distinct features of an audio signal. In addition, in the real environment, there are complex interference factors such as different acoustic events with different lengths and inconsistent background noises, which will affect the recognition accuracy of our system in a practical application. To test the accuracy of the car horns in the audio streaming, we recorded a 30-min audio streaming at the campus gate for testing. We adopted binary classification methods such as fall detection [32], and the following three success criteria could be used to measure its performance.

(1) Recall: The capacity of the system to detect car horns only when they occur.

(13) $$\mathrm{Recall}=\frac{TP}{TP+FN}\times 100\%$$

(2) Precision: The capacity of the system to detect car horns. It is the ratio of true positives to the number of car horns.

(14) $$\mathrm{Precision}=\frac{TP}{TP+FP}\times 100\%$$

(3) F1-score: A harmonic average of model accuracy and recall.

(15) $$F1=\frac{2\times \mathrm{Percision}\times \mathrm{Recall}}{\mathrm{Precision}+\mathrm{Recall}}\times 100\%$$

where *TP* refers to the true positive (i.e., a car horn occurs and the algorithm detects it), FN is a false negative (a car horn occurs but the algorithm does not detect it), and FP is the false positive (a car horn does not occur but the algorithm reports a car horn).

In fact, the audio streaming contains 74 car horns. As shown in Table 9, our system accurately recognized 71 car horns but missed three car horns and incorrectly recognized 16 other sounds as the car horn. There are two reasons for the high false positive. First, the training data and test data are collected in a real environment where complex and variable background noise exist. We artificially checked the audio streaming and found that there are 11 engine sounds, two brake sounds, and two shouts in the 16 FP samples. Their spectral components were similar to the car horn, which is also the cause of the false positive. Second, the training data used in our system were limited, which resulted in finitely pure features of the car horn when we extracted features. With the increase of training data, the samples of false positive will be reduced. We considered that the model of the system has a good performance in sensitivity (95.9%) and precision (81.6%). The complex background noise in the real environment, that is, the low SNR, resulted in an imperfect precision but was sufficient for a real-life application.

#### 4.3.3. The Visualization of Monitoring Platform

**Figure 11:**
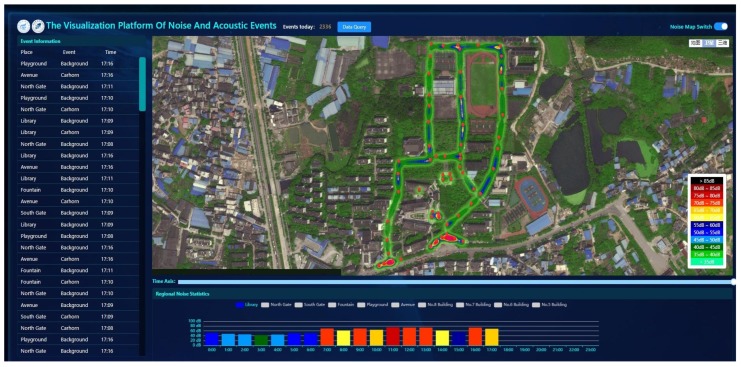
The monitoring platform.

**Figure 12:**
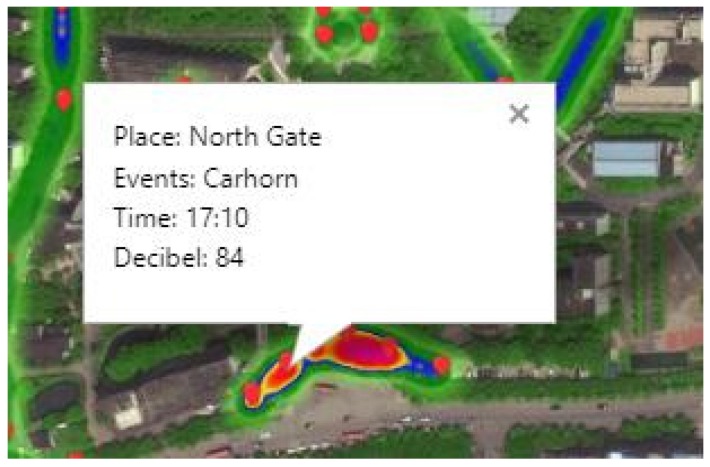
The presentation of acoustic event information.

**Figure 13:**
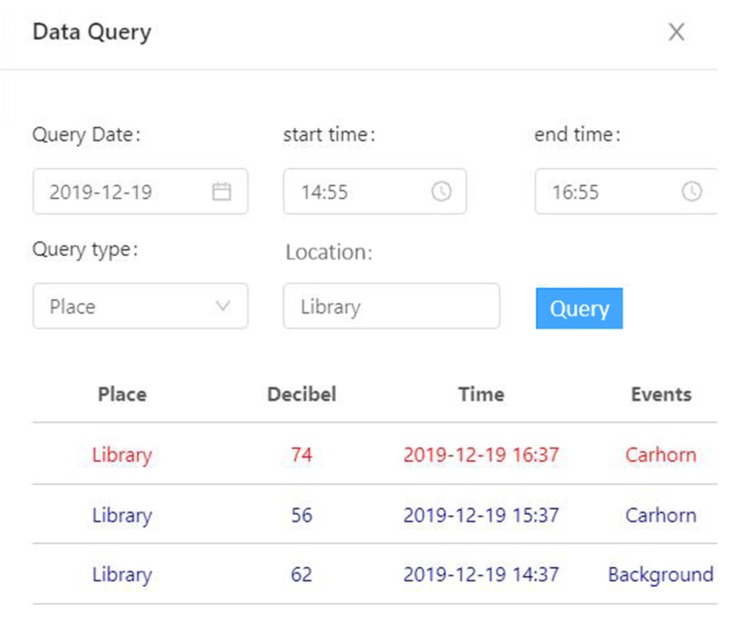
The function of data query.

Finally, we developed a monitoring platform to display the noise map, noise change of sensor nodes, and environmental sound event information. As shown in Figure 11, the 50 sensor nodes are marked on the map in the middle of the platform according to the location of actual deployment. The noise value measured by each sensor node was visually represented by the color circle, and interpolation was performed between two adjacent sensor nodes so that it can form a linear distribution. On the bottom of the platform, a colorful histogram displayed the noise statistics of some important area in a day. We can choose a different area to see the corresponding variation of noise. On the left side of the platform, the column of sound event information displayed the sound event information, including location, event, and time. As shown in Figure 12, the landmark thrived briefly when the system detected an acoustic event of the gate, what is more, the corresponding event information was presented if we clicked it. At the same time, the event information is displayed and recorded in the information column on the left. As shown in Figure 13, a button of data query was set on the top left of the platform, and all the above data can be reviewed by selecting data and time. Users can access the monitoring platform through the URL to view real-time data and historical data.

## 5. Conclusions

In this paper, we proposed a complete conceptual implementation based on the Wireless Acoustic Sensor Network (WASN) for recording, transferring, post-processing, and recognition of noise in order to create and visualize noise maps of acoustic events, as well as to present additional information and noise statistics. We reported the design details, implementation, and real-world deployment of the proposed system. Specifically, we adopted the improved endpoint detection method and compression with ADPCM to reduce the amount of data under the premise of ensuring audio quality and packaged the sensor node for long-term outdoor monitoring. The proposed system not only captures the noise level from each sensor node, but also captures audio data when the noise level reaches a threshold. These data were successfully used for construction of noise map and recognition of acoustic event, presenting the results on a monitoring platform. Most importantly, in Section 4, we reported on the field deployment and analysis of a WASN consisting of 50 sensor nodes in the campus, which indicates that the proposed system has a high accuracy of sound level and well capacity for recognition of the car horn in real life.

In our future works, we will report a larger deployment with our system in a district of our city, combining the improved model of CNNs to automatically classify multiple species of acoustic events, such as shout, crash, explosion. It is of great significance to improving people’s quality of life and ensuring the safety of the living environment. We will research a semi-supervised tensor completion algorithm for inferring noise levels for locations without measurements by sensor nodes. Consequently, the system can be improved by enhancements on both hardware and software.

## Figures and Tables

**Table 1:** The cost of each sensor node.

Description	Material Cost
Acoustic sensor	$12.8
Embedded processing board	$34.3
Wireless transmission module	$28.5
Solar panel	$10.9

**Table 2:** Data information in MySQL (My Structured Query Language).

Num	Time	Coords (latitude, longitude)	IMEI	dB
158	20190702143712	25.289270, 110.343036	0005	56
159	20190702143714	25.288436, 110.343405	0003	49
160	20190702143715	25.289270, 110.342036	0004	50

**Table 3:** The number of data received on the server.

Node Number	The Number of Data Received on the Server	Missing Rate
1	2997	0.10%
2	2995	0.16%
3	2998	0.07%
4	2997	0.10%

**Table 4:** The accuracy of three kinds of sounds with four classification methods.

Methods	Gunshots	Screams	Car Horns
RF	83%	78.5%	77%
KNN	79%	56.3%	86.2%
SVM	80.7%	70.7%	73.1%
CNN	97.3%	88.5%	94%

**Table 5:** Time-consuming of feature extraction and recognition.

Samples Size	Samples Length (ms)	Time-Consuming of Feature Extraction (ms)	Time-Consuming of Recognition (ms)	Time-Consuming of Extraction and Recognition (ms)
10,000	139,120,15	182,100,1	2340	18,233,41

**Table 6:** The number of samples in our dataset.

Species	Car Horn	Background
Train	1160	1052
Test	523	474
C-V	59	53
Total	1742	1579

**Table 7:** Confusion matrix of the CNN model before compression.

	Car Horn	Background
**Car Horn**	491	32
**Background**	10	464

**Table 8:** Confusion matrix of the CNN model after compression.

	Car Horn	Background
**Car Horn**	480	43
**Background**	9	465

**Table 9:** Recognition result with audio steaming.

Metric	Result
TP	71
FP	16
FN	3
Recall	95.9%
Precision	81.6%
F1-score	88.2%

## Abbreviations

The following abbreviations are used in this manuscript:

WASN	Wireless Acoustic Sensor Network
CNNs	Convolutional Neural Networks
GIS	Geographic Information System
3GPP	Third Generation Partnership Project
PCM	Pulse Code Modulation
ADPCM	Adaptive Differential Pulse Code Modulation
CPU	Central Processing Unit
GPU	Graphic Processing Unit
DRAM	Dynamic Random Access Memory
IDE	Integrated Development Environment
API	Application Programming Interface
MFCC	Mel Frequency Cepstrum Coefficient
DCT	Discrete Cosine Transform
ABS	Acrylonitrile Butadiene Styrene
SNR	Signal to Noise Ratio
RF	Random Forest
KNN	K-Nearest Neighbor
SVM	Support Vector Machine
URL	Uniform Resource Locator

## Appendix A

The analysis of $GPRMC sentence.

$GPRMC, <1>, <2>, <3>, <4>, <5>, <6>, <7>, <8>, <9>, <10>, <11>, <12>*hh

<1> UTC Time. Format: Hhmmss

<2> Status. Format: A=Valid, V=Invalid

<3> Latitude. Format: Ddmm.mmmm

<4> N/S hemisphere

<5> Longitude. Format: Dddmm.mmmm

<6> E/W hemisphere

<7> Speed over ground

<8> Course over ground

<9> UTC Date. Format: Ddmmyy

<10> Magnetic variation

<11> Direction of magnetic variation

<12> Checksum
